# Structural divergence of chromosomes between malaria vectors *Anopheles lesteri* and *Anopheles sinensis*

**DOI:** 10.1186/s13071-016-1855-0

**Published:** 2016-11-25

**Authors:** Jiangtao Liang, Biao Cheng, Guoding Zhu, Yun Wei, Jianxia Tang, Jun Cao, Yajun Ma, Maria V. Sharakhova, Ai Xia, Igor V. Sharakhov

**Affiliations:** 1 Department of Entomology, Nanjing Agricultural University, Nanjing, China; 2 Key Laboratory of National Health and Family Planning Commission on Parasitic Disease Control and Prevention, Jiangsu Provincial Key Laboratory on Parasite and Vector Control Technology, Jiangsu Institute of Parasitic Diseases, Wuxi, Jiangsu Province China; 3 Department of Tropical Infectious Diseases, Faculty of Tropical Medicine and Public Health, Second Military Medical University, Shanghai, 200433 China; 4 Department of Entomology, Fralin Life Science Institute, Virginia Tech, Blacksburg, VA USA; 5 Laboratory for Ecology, Genetics and Environmental Protection, Tomsk State University, Tomsk, Russia

**Keywords:** Cytogenetic map, Chromosomal inversions, *Anopheles lesteri*, *Anopheles sinensis*, Gene order, Arm homology, Polytene chromosomes

## Abstract

**Background:**

*Anopheles lesteri* and *Anopheles sinensis* are two major malaria vectors in China and Southeast Asia. They are dramatically different in terms of geographical distribution, host preference, resting habitats, and other traits associated with ecological adaptation and malaria transmission. Both species belong to the *Anopheles hyrcanus* group, but the extent of genetic differences between them is not well understood. To provide an effective way to differentiate between species and to find useful markers for population genetics studies, we performed a comparative cytogenetic analysis of these two malaria vectors.

**Results:**

Presented here is a standard cytogenetic map for *An. lesteri*, and a comparative analysis of chromosome structure and gene order between *An. lesteri* and *An. sinensis*. Our results demonstrate that much of the gene order on chromosomes X and 2 was reshuffled between the two species. However, the banding pattern and the gene order on chromosome 3 appeared to be conserved. We also found two new polymorphic inversions, 2Lc and 3Rb, in *An. lesteri*, and we mapped the breakpoints of these two inversions on polytene chromosomes.

**Conclusions:**

Our results demonstrate the extent of structural divergence of chromosomes between *An. lesteri* and *An. sinensis*, and provide a new taxonomic cytogenetic tool to distinguish between these two species. Polymorphic inversions of *An. lesteri* could serve as markers for studies of the population structure and ecological adaptations of this major malaria vector.

**Electronic supplementary material:**

The online version of this article (doi:10.1186/s13071-016-1855-0) contains supplementary material, which is available to authorized users.

## Background

*Anopheles sinensis* Wiedemann and *Anopheles lesteri* Baisas & Hu, 1936 (syn. *An. anthropophagus*) are members of the *Anopheles hyrcanus* group. Due to their identical morphological characteristics, they have been considered cryptic species [1]. The earliest studies in the 1960s identified two egg types of *An. sinensis* in China [2]. Since then, the egg type with a wide deck has been assigned to *An. sinensis*, while the taxonomic status and specific name for the narrow egg type remains questionable. Xu & Feng [3] initially proposed that the narrow egg type of *An*. s*inensis* represents a subspecies of *An. lesteri*, and named it *An. lesteri anthropophagus*. Later, Ma [4] elevated *An. anthropophagus* to the species level. However, recent studies of the internal transcribed spacer 2 (ITS2) of the ribosomal DNA (rDNA) have provided evidence that *An. anthropophagus* is a junior synonym of *An. lesteri*, leading to a resurrection of the species name *An. lesteri* Baisas & Hu, 1936 [4–6]. Therefore, the previously recognized *An. anthropophagus* and *An. lesteri* in China are considered the same species.

Despite being morphologically indistinguishable, members of the *An. hyrcanus* group, *An. lesteri* and *An. sinensis* differ in their geographical distribution, host preference, resting habitats, and other features important for malaria transmission [7]. *Anopheles sinensis* is the most widespread species, with a range that is continuous throughout 29 provinces and regions in China. *Anopheles lesteri* is mostly sympatric with *An. sinensis* but is limited to central China, south of 33°N [7]. *Anopheles lesteri* has been established as the primary vector of *Plasmodium vivax* malaria in central China because of the higher human blood index of *An. lesteri* and its close association with malaria outbreaks. In central China, most malaria outbreaks appeared in areas where *An. lesteri* was present. The regions containing *An. lesteri* have suffered more serious malaria epidemics than those areas where the only vector was *An. sinensis* [8]. In addition, *An. lesteri* naturally transmits *P. falciparum* [8, 9] and also is a major vector of a filarial worm, *Brugia malayi*, in China [10, 11]. *An. sinensis* is more zoophilic and has significantly lower human-biting rate, human blood index, infection rate, and entomological inoculation rate with *P. vivax* than *An. lesteri* [9]. For decades it has been considered a secondary malaria vector. However, the importance of *An. sinensis* as the *P. vivax* malaria vector in areas cultivated for rice (*Oryza sativa* L.) cannot be ignored. Recent evidence indicates that *An. sinensis* was responsible for the outbreak of the vivax malaria in central China in 2006 [12, 13].

The availability of well-developed polytene chromosomes in the salivary glands of *An. lesteri* and *An. sinensis* makes these species suitable for cytogenetic studies [14, 15]. Before the high-resolution cytogenetic photomap of *An. sinensis* was published in 2014 [14], several chromosomal maps were developed for this species [15–19] and used to distinguish among sibling species within the *An. hyrcanus* group. However, because most of the published maps are drawn manually, their resolution is low, which has introduced subjectivity into the interpretation of chromosome banding patterns. In contrast to the previously drawn maps, the standard photomap for *An. sinensis*, constructed from high-resolution chromosome images [14], provides more detailed banding patterns, making it more suitable for genome mapping and other studies.

In this study, a detailed cytogenetic photomap was developed for the Asian malaria vector *An. lesteri*. A comparative analysis of the chromosome banding patterns between *An. lesteri* and *An. sinensis* demonstrated a limited pattern identity between chromosomes X and 2. Gene mapping, using fluorescent *in situ* hybridization (FISH), further established whole-arm homology between the two species. A gene order comparison determined rearrangements of the X, 2R, and 2L chromosomes, while arms 3R and 3L were more conserved between the two species. We also mapped two new polymorphic inversions: 2Lc in field-collected *An. lesteri* specimens from Hainan, and 3Rb in the Wuxi laboratory strain of *An. lesteri*. These could serve as markers for distinguishing between the two species and for population genetics and ecological studies of *An. lesteri*.

## Methods

### Mosquito strains and chromosome preparation

The Wuxi laboratory strain (Jiangsu Institute of Parasitic Diseases, Wuxi, China) and a field strain of *An. lesteri* collected from Hainan were used in this study. The Wuxi strain of *An. lesteri* has been maintained in the insectary of the Key Technical Laboratory for Prevention and Control of Parasitic Diseases of the Ministry of Health (MOH) in the Jiangsu Institute of Parasitic Diseases (JIPD), Wuxi, China, for over 30 years. The salivary glands were dissected from early fourth-instar larvae of *An. lesteri* and were used for obtaining polytene chromosome preparations as previously described [14]. The quality of polytene chromosomes was examined with an Olympus BX43 phase-contrast microscope (Olympus Corp., Tokyo, Japan). Chromosome preparations with clear banding patterns were placed in liquid nitrogen for several minutes, and then cover slips were removed and slides were dehydrated in 50%, 70%, 90% and 100% ethanol for imaging and *in situ* hybridization. Chromosomes were imaged with an Olympus BX43 microscope with a DP72 digital camera and CellSens imaging software (Olympus Corp.). The best 50 of approximately 100 images were used to construct a cytogenetic map for *An. lesteri*, using Adobe Photoshop software as previously described [20].

### Fluorescence *in situ* hybridization

The Genome sequences were acquired from the *An. gambiae* PEST strain (https://www.vectorbase.org/organisms/anopheles-gambiae/pest) and *An. sinensis* (https://www.vectorbase.org/organisms/anopheles-sinensis/sinensis). The PCR primers were designed using the Primer3 Program [21], based on the above genome sequences from *An. gambiae* and *An. sinensis*. Genomic DNA of *An. lesteri* was extracted from live fourth-instar larvae, using a DNeasy Blood & Tissue Kit (Qiagen GmbH, Hilden, Germany), and utilized as a template for PCR.

After the standard PCR procedures, amplified PCR products were loaded on agarose gel and cut for purification using a QIAquick Gel Extraction Kit (Qiagen). The DNA fragments were labeled with either Cy3.5-AP3-dUTP or Cy5.5-AP3-dUTP (GE Healthcare UK Ltd, Chalfont StGiles, UK), using a Random Primed DNA Labelling Kit (Roche Applied Science, Penzberg, Germany). *In situ* hybridization was performed using a previously described method [20]. Fluorescent signals were detected and recorded with a Zeiss LSM 710 laser scanning microscope (Carl Zeiss Microimaging GmbH, Oberkochen, Germany), and placed on the polytene chromosome map of *An. lesteri*.

## Results and discussion

### A polytene chromosome photomap for *An. lesteri*

**Fig. 1:**
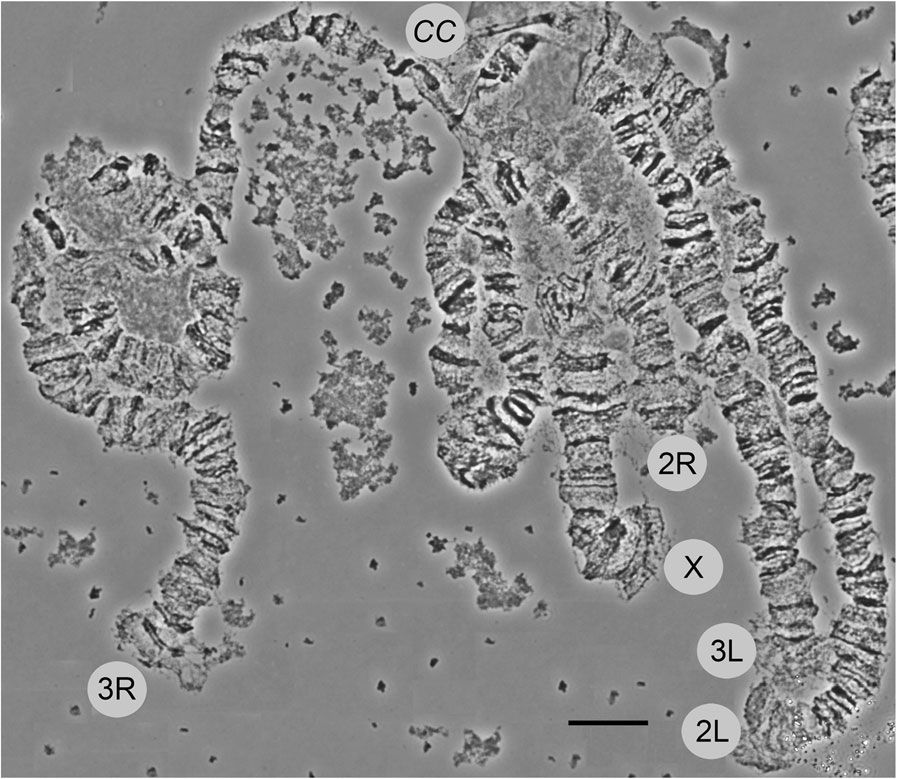
Photomicrograph of the polytene chromosomes of *Anopheles lesteri*. X, 2R, 2L, 3R and 3L represent chromosomal arms and CC indicates the chromocenter. *Scale-bar*: 10 µm

**Fig. 2:**
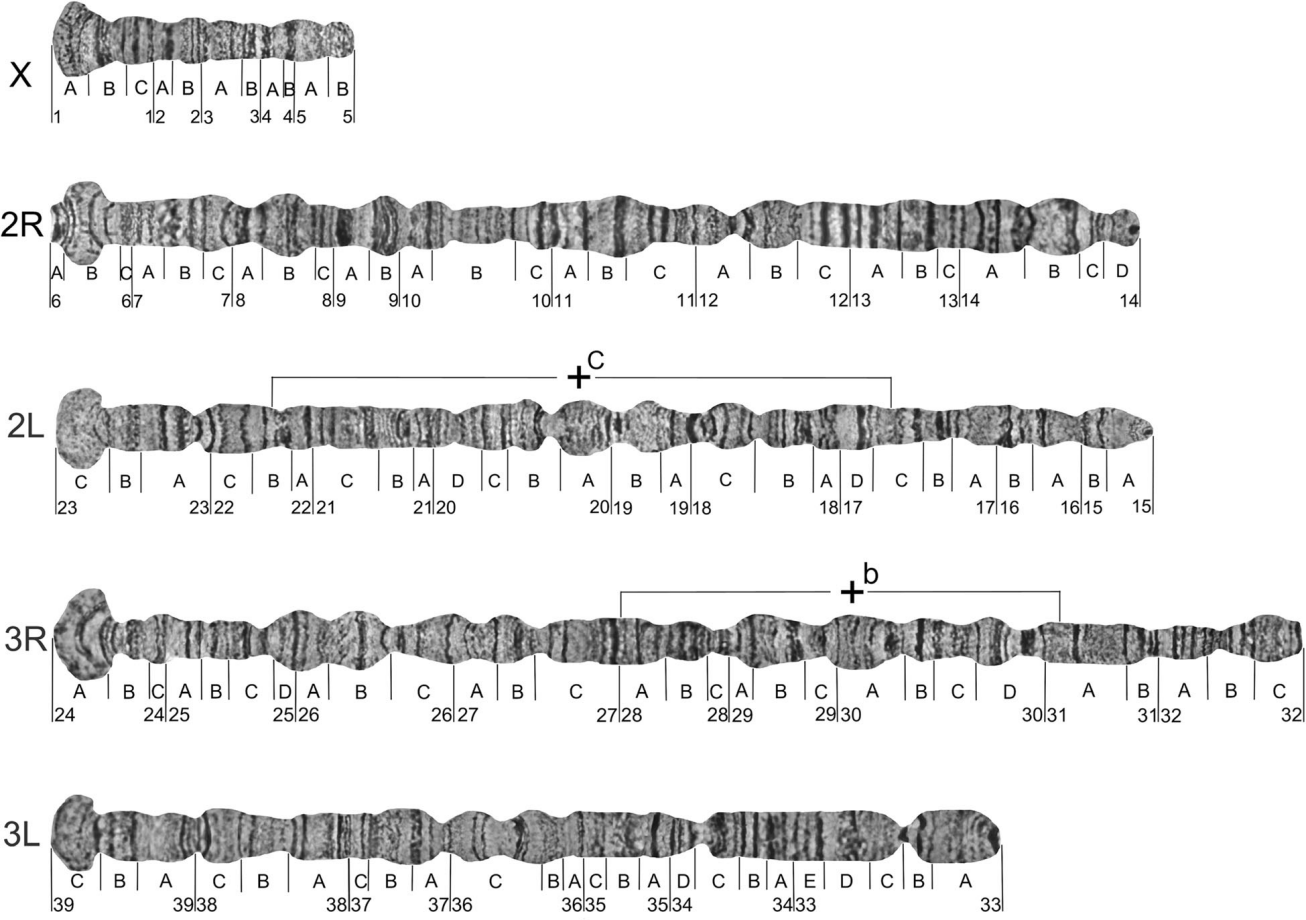
A standard cytogenetic map for *Anopheles lesteri.* Numbered divisions and lettered subdivisions are shown below the chromosomes. Brackets indicate the positions of the polymorphic inversions 2Lc and 3Rb

The polytene chromosome complement in salivary glands of *An. lesteri* consists of three chromosomes (X, 2, and 3). The X chromosome is represented by one arm, while chromosomes 2 and 3 have two arms. Chromosomal arms usually form the chromocenter, by joining their pericentromeric regions (Fig. 1). Polytene chromosomes with clear banding patterns from the salivary glands of *An. lesteri* were analyzed and utilized to develop a standard photomap. The chromosomal map contains five elements: the shortest X chromosome, the longest 3R arm, and then similarly sized 2R, 2L, and 3L arms. Chromosome elements of *An. lesteri* were subdivided into 39 numbered divisions and 116 lettered subdivisions, by analogy with the chromosome map of *An. sinensis* [14] (Fig. 2).

The landmarks which could be used for arm recognition were as follows. In addition to its short length, the X chromosome of *An. lesteri* could be recognized by telomeric region 1A, the end of which has a “flared fan” shape with two thin granulated bands (Fig. 2). The centromeric end of the X chromosome has a bulbous, granulated region, 5B, with an unclear banding pattern. Autosome 2 consists of two arms of almost equal length (Fig. 2). The telomeric end of 2R has three narrow bands at the tip, followed by a large puffy area in subdivision 6B. A wide dark band and a bright inter-band in region 9A, and a puffy region 9B with three double bands, could also be used as landmarks for the 2R arm. A dark double band in region 11B and two dark bands followed by four thin bands in region 11C provide additional landmarks for the middle part of the 2R arm. The centromeric end of the 2R arm has a small puffy region, 14D, with a dark band on the very end. In contrast to the bulb-shaped and striped telomeric end of the 2R arm, the 2L arm has a flared, bright, granulated telomeric end, followed by four dark bands in region 23A. A large, bright, puffy region, 18C, with six dark bands, provides supporting information that this is the 2L arm. The pericentromeric area is easily recognized by a dark band with a low level of polyteny in region 15A, and two bands in region 15B.

Chromosome 3 has arms that are unequal in length. The 3R arm is the longest among them (Fig. 2). The telomeric area of 3R has a large region with a flared fan shape and a dark double band inside followed by three thin bands at the beginning of region 24B. Another consistent feature of 3R is the presence of two large puffy areas in regions 25D–26B; one contains three dark bands and the other has one dark double band in the middle. The presence of one dark band in regions 31A, surrounded by bright granulated areas on both sides, could be considered an additional landmark for this arm. Close to the centromere, the 3R arm is composed of a series of continuous dark bands in regions 31B–32B, which makes this region look striped.

The 3L arm is slightly shorter than the chromosome 2 arms. The telomeric end of 3L is bright and flared, as the 2L arm, but can be easily distinguished by the presence of three dark bands in the 39B-C region. Two puffy areas in the 36B-C region, one with close three dark bands and the other with two dark bands in the middle, could also be considered an important landmark of the 3L arm. Closer to the centromere, this arm contains an apparent constriction in the 33B-C region, with a dark band in the middle. The centromeric end of the 3L arm consists of a large dark band, which is absent in the centromeric areas of the other chromosomal arms.

### Polymorphic inversions in *An. lesteri*

**Fig. 3:**
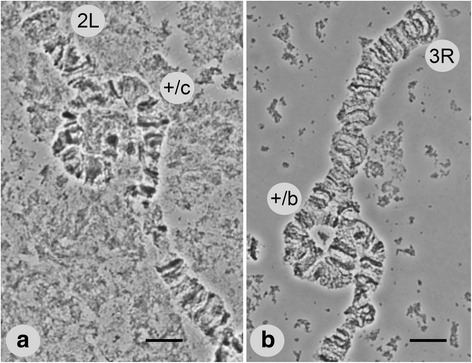
Heterozygote loops of polymorphic inversions 2Lc (**a**) and 3Rb (**b**) of *An. lesteri*. Positions of the loops are indicated as +/c and +/b. *Scale-bars*: 10 µm

The results of our study of field-collected and laboratory strains demonstrated the presence of two polymorphic inversions in chromosome arms 2L and 3R of *An. lesteri*. A polymorphic inversion in a heterozygote state on the 2L (2Lc) arm was found in 20 of 76 samples from the field strain of *An. lesteri* collected in Hainan (Fig. 3). This inversion was not present in the Wuxi laboratory strain. The distal and proximal breakpoints of the 2Lc inversion were mapped to the regions 22B and 17C of 2L (Fig. 2). Our results revealed a landmark for recognizing this inversion. In the case of the standard arrangement of chromosome arm 2L (+^c^/+^c^), the banding pattern was arranged as one puff with four dark bands followed by three thin bands (18C-B) near the proximal part of chromosome. In the inverted chromosome (2Lc/c), the bands appeared as three thin bands, plus one puff containing four dark bands near the distal part of the chromosome.

We also discovered another polymorphic inversion on 3R (3Rb), but only in the Wuxi laboratory strains of *An. lesteri*, with a frequency of heterozygote arrangement of about 17%. We localized the breakpoints to regions 27C and 31A on 3R (Fig. 2). The landmark of the standard 3R arrangement (+^b^/+^b^) was four closely located bands followed by a dark triple band located in the proximal position (30D). In an inverted chromosome (3Rb/b), this specific banding pattern is at a distal position.

Inversion polymorphisms are commonly used as tools in mosquito taxonomy, ecology, and population genetics. Data from a previous study indicated that adaptations of mosquitoes to various environments are often associated with the composition and frequency of polymorphic inversions [22, 23]. Despite the importance of *An. lesteri* for malaria transmission in Asia, inversion polymorphisms have not been investigated.

In this study, we identified the polymorphic inversion 2Lc (17C-22B) from a field population in Hainan, and 3Rb (27C-31A) from Wuxi laboratory strains of *An. lesteri*. We previously published one polymorphic inversion (3Ra) on chromosome arm 3R of *An. sinensis*, and mapped the breakpoints to regions 28A-31A [14]. However, without genome sequencing data, we could not analyze the molecular structures of inversion breakpoints and could not determine if the 3Rb inversion in *An. lesteri* and 3Ra in *An. sinensis* are the same. Nevertheless, the inversion polymorphism reported here for *An. lesteri* would help our understanding of epidemiological and ecological heterogeneities in this malaria vector.

### Structural divergence of chromosomes and gene order comparison between *An. lesteri* and *An. sinensis*

**Fig. 4:**
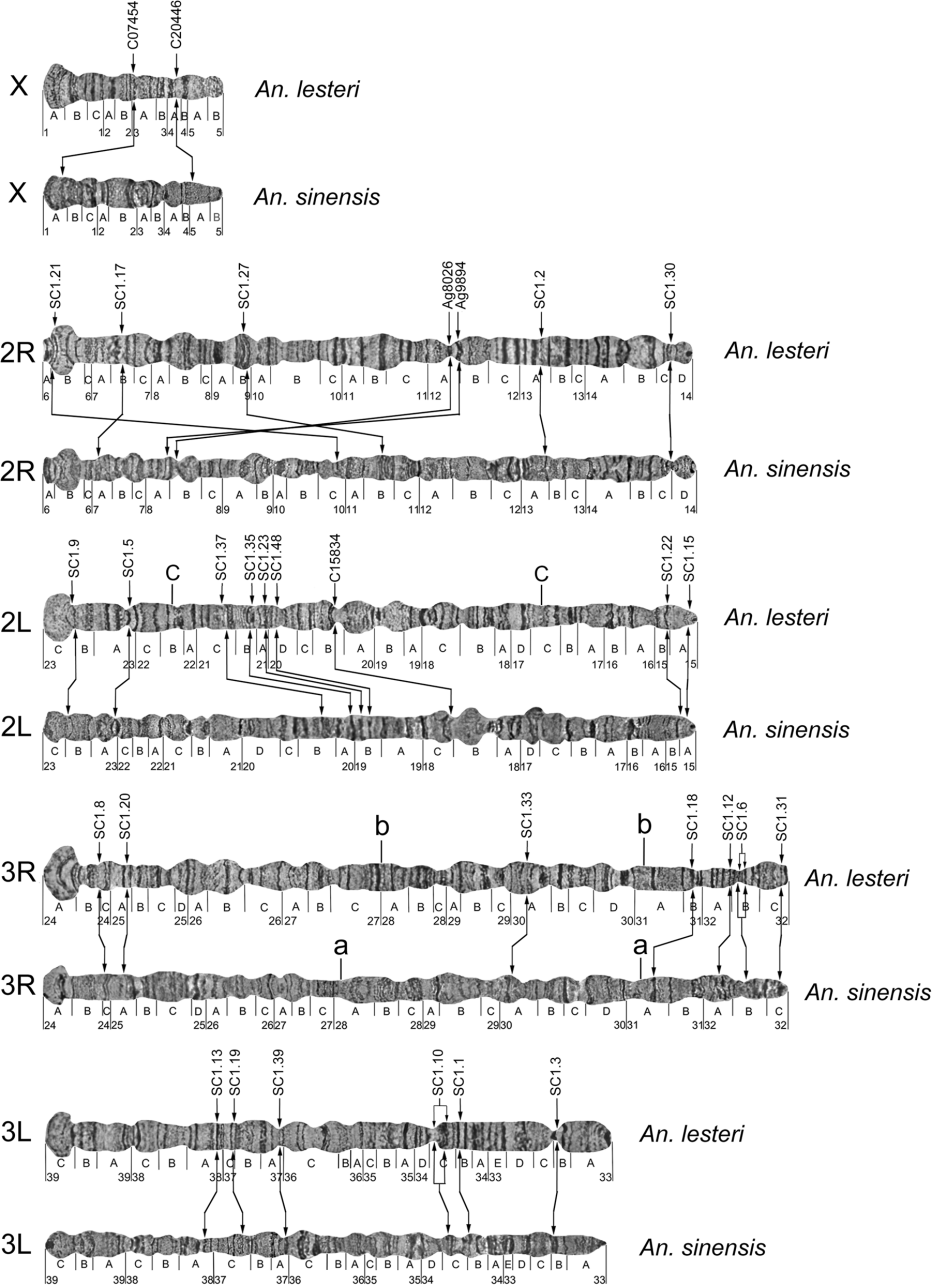
Comparison of DNA probe locations and chromosome structures between *An. lesteri* and *An. sinensis*. Arrows indicate positions of the probes on chromosomes of the two species

**Fig. 5:**
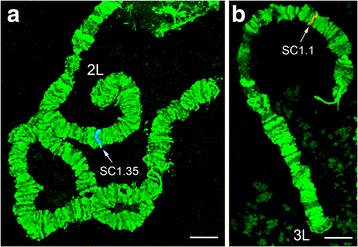
Fluorescence *in situ* hybridization results of DNA probes SC1.35 and SC1.1. Arrows show the positions of signals produced by probes (**a**) and SC1.1 (**b**). Blue signal were labeled with Cy5.5 and red were labeled with Cy3.5 dyes. *Scale-bars*: 10 µm

Banding pattern comparisons based on cytogenetic maps of *An. lesteri* and *An. sinensis* [14], and gene order analysis using FISH of 31 PCR-amplified DNA probes on chromosomes of both species, enabled us to perform a comparative cytogenetic study (Fig. 4). To amplify DNA probes for FISH, two pairs of PCR primers (Ag8026 and Ag9894) were designed, based on the *An. gambiae* PEST strain DNA sequences available from the Vector Base (https://www.vectorbase.org/organisms/anopheles-gambiae). Primers for 27 probes were inferred from the genome sequence of *An. sinensis* (https://www.vectorbase.org/organisms/anopheles-sinensis/sinensis). An additional three pairs of PCR primers (C20446, C07454, and C15834) were developed based on our sequence database (Additional file 1: Table S1). All DNA probes yielded unique signals on the polytene chromosomes of *An. lesteri* and *An. sinensis* (Table 1). Two examples of FISH, with one clear signal on each, are shown in Fig. 5.

**Table 1:** DNA probes used for *in situ* hybridization with the *An. lesteri* and *An. sinensis* polytene chromosomes

	Clone name	Source	Location in *An. lesteri*	Location in *An. sinensis*
1	C20446	Additional file 1: Table S1	X:4A	X:1A
2	C07454	Additional file 1: Table S1	X:3A	X:5A
3	SC1.21	supercont1.21	2R:6A	2R:10C
4	SC1.17	supercont1.17	2R:7B	2R:7A
5	SC1.27	supercont1.27	2R:9B	2R:11B
6	Ag8026	AGAP008026	2R:12A	2R:8B
7	Ag9894	AGAP009894	2R:12A	2R:8A
8	SC1.2	supercont1.2	2R:13A	2R:13A
9	SC1.30	supercont1.30	2R:14C	2R:14C
10	SC1.9	supercont1.9	2L:23C	2L:23C
11	SC1.5	supercont1.5	2L:23A	2L:23A
12	SC1.37	supercont1.37	2L:21C	2L:20B
13	SC1.35	supercont1.35	2L:21B	2L:20A
14	SC1.23	supercont1.23	2L:21A	2L:19B
15	SC1.48	supercont1.48	2L:20D	2L:19B
16	C15834	Additional file 1: Table S1	2L:20B	2L:18C
17	SC1.22	supercont1.22	2L:15B	2L:15B
18	SC1.15	supercont1.15	2L:15A	2L:15A
19	SC1.8	supercont1.8	3R:24B	3R: 24B
20	SC1.20	supercont1.20	3R:25A	3R:25A
21	SC1.33	supercont1.33	3R:30A	3R:30A
22	SC1.18	supercont1.18	3R:31B	3R:31A
23	SC1.12	supercont1.12	3R:32A	3R:32A
24	SC1.6	supercont1.6	3R:32B	3R:32B
25	SC1.31	supercont1.31	3R:32C	3R:32C
26	SC1.13	supercont1.13	3L:38A	3L:38A
27	SC1.19	supercont1.19	3L:37C	3L:37C
28	SC1.39	supercont1.39	3L:37A	3L:37A
29	SC1.10	supercont1.10	3L:34C	3L:34C
30	SC1.1	supercont1.1	3L:34B	3L:34B
31	SC1.3	supercont1.3	3L:33B	3L:33B

By comparing the physical locations of the probes in *An. lesteri* and *An. sinensis* (Table 1), we demonstrated that all five arms are homologous between the two species (X = X; 2R = 2R; 2L = 2L; 3R = 3R; 3L = 3L). When we further analyzed the physical locations of DNA probes and compared the banding patterns on five chromosomal arms (Fig. 4), we found that gene locations on the sex chromosome X and autosome 2R were reshuffled. The chromosomal structure showed limited similarity between the two species. Chromosome arm 2L, and particularly arms 3R and 3L, demonstrated relatively consistent gene orders and had some similarities in their banding patterns. Cytogenetic analyses identified specific landmarks on each chromosomal arm that could be used for species recognition.

We hybridized only two probes on the X chromosomes of the two species. These probes, C07454 and C20446, were mapped to different X chromosome regions of the two species: subdivisions 3A and 4A in *An. lesteri* and 5A and 1A in *An. sinensis* (Fig. 4), suggesting the existence of rearrangements between the species.

The banding patterns of X chromosomes had no recognizable similarity between the two species, implying the presence of multiple chromosomal inversions that reshuffled the order of the bands. As a result of significant order reshuffling, the boundaries of the inversions remained uncertain, based on the cytogenetic analysis. Telomeric and centromeric areas of the X chromosomes in the two species could be easily used for species diagnostics. The telomeric end in *An. sinensis* starts with two dark, granulated bands in region 1A, while, in *An. lesteri*, it begins with a bright, granulated, flared end. Secondly, the pericentromeric region 5B ends with a clear dark band in *An. sinensis*, but a bright granulated bulb-shaped area in *An. lesteri*.

Analysis of the positions of seven markers mapped on chromosome arm 2R also identified multiple rearrangements (Fig. 4) between the two species. Using these markers and the Genome Rearrangements in Man and Mouse (GRIMM) program [24], we calculated the minimum number of fixed inversions on arm 2R between *An. lesteri* and *An. sinensis*. The orders of the markers were 1, 2, 3, 4, 5, 6 and 7 in *An. lesteri* and 2, 5, 4, 1, 3, 6 and 7 in *An. sinensis*. When we used the GRIMM program, without assuming directionality of the markers (unsigned option), there were three calculated inversions. When we used the signed option to perform a pairwise analysis, the number of rearrangements increased to five. The banding patterns of these arms significantly differed between the two species.

The most reliable landmarks to distinguish between these arms in the two species are the following. First, six large bands in the middle of arm 2R in regions 10C-11C in *An. lesteri* were not present in the middle of this arm in *An. sinensis*. The puffy region, 9B, with three double bands in *An. lesteri* (the chromosome location of marker SC1.27), changed its position on the *An. sinensis* chromosome, where it was located in the inverted position in the middle of the arm in region 11B. Two dark bands in region 12A in *An. lesteri* (the chromosome locations of markers Ag8026 and Ag9894) could be traced in region 8A of *An. sinensis*, also an inverted order. Telomeric areas of *An. lesteri* and *An. sinensis* were similar to each other, which might suggest that marker SC1.21 was transferred to a new position, not by an inversion, but by a transposition.

The analysis of the order of nine probes on arm 2L for the two species revealed no order reshuffling. However, the distance between the markers SC1.5 and SC1.37 in *An. sinensis* was twice that of *An. lesteri*, suggesting the presence of rearrangements in 2L. These rearrangements moved four areas with puffs from the middle of arm 2L (region 18C-20B) in *An. lesteri* to the centromeric regions 17C-18C in *An. sinensis*. This difference could be used to distinguish between the 2L arms of the two species.

The positions of seven and six DNA probes in arms 3R and 3L, respectively, demonstrated no gene order reshuffling between *An. lesteri* and *An. sinensis*. Although the banding pattern of chromosome 3 was similar between the two species, *An. lesteri* could be distinguished by a flared 3L telomere, and *An. sinensis* could be identified based on a narrow, dark telomeric band. Another specific feature of the 3L arm in *An. lesteri* was constriction in region 33B_C, which was absent in *An. sinensis*. The landmark in the *An. lesteri* 3R arm was four thin dark bands followed by a dark triple band in the region 30D, which was absent in *An. sinensis*.

To investigate the pattern of chromosome rearrangements over larger evolutionary distances, we conducted BLAST searches of homologous sequences in the *An. atroparvus* [25] and *An. gambiae* [26] genomes. *Anopheles atroparvus* is the only member of the *An. maculipennis* complex with a sequenced genome. Some of the genomic scaffolds of *An. atroparvus* were recently mapped to chromosomes of this species [25, 27]. These data enabled us to assign the *An. lesteri* probes to chromosomal arms of *An. atroparvus* (Table 2).

**Table 2:** Genomic locations of the DNA probes in *An. atroparvus*, *An. sinensis* and *An. gambiae*. The locations were acquired using https://www.vectorbase.org/blast against genomic scaffold sequences of *Anopheles atroparvus* EBRO strain, *Anopheles gambiae* PEST strain, and *Anopheles sinensis *China strain. The e-values are indicated in parentheses after the locations

	Clone name	Source	Location in *An. atroparvus*	Location in *An. sinensis*	Location in *An. gambiae*
1	C20446	Additional file 1: Table S1	KI421895: 3842010–3842293(3e^-128^); KI421886: 1702493– 1702773(8e^-98^)	AS2_scf7180000695491: 102496–102779(2e^-144^)	X:533418–533760(6e^-107^)
2	C07454	Additional file 1: Table S1	KI421888: 9432816–9432478(1e^-76^)	AS2_scf7180000695709:907649–907989(2e^-175^)	2L:47798210–47798610(1e^-96^)
3	SC1.21	supercont1.21	2R:KI421882: 9912851–10045608(0)	AS2_scf7180000696054: 1195628–1428863 (0)	3R:10,114,393–10,249,464 (0)
4	SC1.17	supercont1.17	KI421890: 1035344–1389232(0)	AS2_scf7180000695920: 44079–328299 (0)	3R:38705671–38,709,129(0)
5	SC1.27	supercont1.27	KI421890: 4179223–4195140(0)	AS2_scf718000069604:488088–637274(0)	3R:26871040–26944051(0)
6	Ag8026	AGAP008026	KI421890: 6995505–6995742(5e^-91^)	AS2_scf7180000696017: 292678–292915(3e^-81^)	3R:4,499,175–4,507,999(0)
7	Ag9894	AGAP009894	2R:KI421882: 15579762–15579934(2e^-52^)	AS2_scf7180000696027: 184208–184042(2e^-53^)	3R:44,903,740–44,912,595(0)
8	SC1.2	supercont1.2	KI421900: 2361929–2838297(0)	AS2_scf7180000696013: 1377801–1404489(0) AS2_scf7180000695708: 122951–293931(0)	3R:49911437–50,354,787(0)
9	SC1.30	supercont1.30	KI421900: 1492320–1595685(0)	AS2_scf7180000696012: 752618–1009731(0)	2L:5,452,203–5,453,482(0)
10	SC1.9	supercont1.9	2L:KI421884: 10941708–11186226(0)	AS2_scf7180000695483: 403208–410683(0)	2L:47,782,863–48,943221(0)
11	SC1.5	supercont1.5	2L:KI421884: 77975–136.076(0) KI421916: 285574–290011(0)	AS2_scf7180000696060: 1083588–1123553(0)	2L:33212653–33,787,298(0)
12	SC1.37	supercont1.37	KI421886: 11137039–11199731(0)	AS2_scf7180000696049: 2284815–2306994(0) AS2_scf7180000696129: 257010–238213(0)	2L:36,690,171–36,694,229(0)
13	SC1.35	supercont1.35	2L:KI421886: 9346759–9417257(0)	AS2_scf7180000696058: 981634–993925(0)	2L:27,916,638–27,918,874(0)
14	SC1.23	supercont1.23	KI421891: 7010523–7018018(0)	AS2_scf7180000695983: 404330–428660(0)	2L:11,396,721–11,400,344(0)
15	SC1.48	supercont1.48	KI421891: 6344135–6346162(0)	AS2_scf7180000695983: 1052702–1083490(0)	2L:10,641,721–10,643,463(0)
16	C15834	Additional file 1: Table S1	2L:KI421886: 1283313–1283772(1e^-^^167^)	AS2_scf718000069587: 127420–127901(0)	2L:26,996,740–26,997,260(9e^-133^)
17	SC1.22	supercont1.22	2L:KI421886: 998049–1001402(0)	AS2_scf7180000690255: 166481–193533(0)	2L:14,179,779–14,182,651(0)
18	SC1.15	supercont1.15	KI421924: 86788–88570(0)	AS2_scf7180000695974: 485746–503624(0)	2L:2,429,065–2,431,713(0)
19	SC1.8	supercont1.8	KI421888: 4420609–4428976(0)	AS2_scf7180000695742: 846781–876896(0)	2R:11,955,880–11,963,441(0)
20	SC1.20	supercont1.20	KI421897: 3217947–3221214(0)	AS2_scf7180000695544: 217804–260601(0)	2R:40,388,954–40,391,204(0)
21	SC1.33	supercont1.33	KI41906: 789097–795883(0)	AS2_scf7180000695987: 203015–213211(0)	2R:9,258,761–9,262,721(0)
22	SC1.18	supercont1.18	KI421885: 5623993–5627430(0)	AS2_scf7180000691904: 29065–60946(0)	2R:53,122,916–53,125,899(0)
23	SC1.12	supercont1.12	KI421883: 793616–798090(0)	AS2_scf7180000696020: 346403–391124(0)	2R:25,078,045–25,082,179(0)
24	SC1.6	supercont1.6	KI421885: 9679627–9684068(0)	AS2_scf7180000696131: 1492568–1550122(0)	2R:51,006,383–51,010,426(0)
25	SC1.31	supercont1.31	KI421908: 618377–622446(0)	AS2_scf7180000695941: 606638–632554(0)	2R:59,756,833–59,757,717(1e^-156^)
26	SC1.13	supercont1.13	KI421893: 6014521–6018625(0)	AS2_scf7180000696026: 562865–534327(0)	3L:8,096,738–8,100,402(0)
27	SC1.19	supercont1.19	KI421899: 300613–303079(0)	AS2_scf7180000695747: 106108–123837(0)	3L:26,049,483–26,051,315(0) X:15,344,337–15,345,182(2e^-162^)
28	SC1.39	supercont1.39	KI421893: 834871–838718(0)	AS2_scf7180000695549: 106490–85123(0)	3L:18,123,024–18,126,350(0)
29	SC1.10	supercont1.10	KI421901: 2592790–2596255(0)	AS2_scf7180000695556: 1356231–1372024(0)	3L:32,619,834–32,623,094(0)
30	SC1.1	supercont1.1	KI421887: 668313–671209(0)	AS2_scf7180000696001: 472078–478139(0)	3L:19,636,991–19,638,260(0)
31	SC1.3	supercont1.3	KI421913: 114687–120327(0)	AS2_scf7180000695959: 371603–412620(0)	3L:3,313,404–3,318,603(0)

There was good correspondence in arm assignment between *An. lesteri* and *An. atroparvus*. The close association of arm assignments in *An. lesteri*, *An. atroparvus* and *An. sinensis* support their close relationships, as they belong to the subgenus *Anopheles*. In contrast, the 2R arm of *An. lesteri* corresponded to the 3R arm in *An. gambiae*, and *vice versa*, indicating a whole-arm translocation between the species. Thus, our results provide further support for evidence of a whole-arm translocation between subgenera *Anopheles* and *Cellia* [28], to which *An. gambiae* belongs.

## Conclusions

In this study, we constructed a standard cytogenetic map for the Asian malaria vector *An. lesteri*. In addition, we identified polymorphic inversions: 2Lc, from a field population in Hainan, and 3Rb on the 3R chromosome from the Wuxi laboratory strain of *An. lesteri*. The inversion breakpoints were localized to subdivisions 22B and 17C of 2L, and 27C and 31A of 3R. Polymorphic inversions are useful markers for studying the population genetics of mosquitoes. These results provide the foundation for a better understanding of the epidemiological and ecological roles of polymorphic inversions in *An. lesteri*.

By comparing the physical locations of 31 probes of *An. lesteri* and *An. sinensis*, and the banding patterns of polytene chromosomes, we found that all five chromosome arms were homologous for *An. lesteri* and *An. sinensis*. We demonstrated that the chromosome arms X, 2R, and 2L were rearranged between the two species, because of the presence of fixed inversions. Chromosome structures of the 3R and 3L arms were more similar, and gene orders were conserved, between *An. lesteri* and *An. sinensis*.

Our results provide reliable cytogenetic information for discriminating *An. lesteri* and *An. sinensis*, and a foundation for understanding the genetic content associated with species-specific ecological adaptation and vectorial capacity. Cytogenetic and physical maps for *An. lesteri* and *An. sinensis* would serve as convenient outgroups for phylogenetic reconstruction, based on fixed inversions in other mosquitoes of the subgenus *Anopheles*, such as the *An. maculipennis* complex.

## Additional file

Additional file 1: Table S1. The sequences of DNA probes from *An. sinensis* Shanghai Chinese strain. (DOCX 15 kb)
