# Supplementary material for: Structural divergence of chromosomes between malaria vectors Anopheles lesteri and Anopheles sinensis
Source: Parasit Vectors. 2016 Nov 25;9:608. doi: 10.1186/s13071-016-1855-0 (PMC5124334; doi:10.1186/s13071-016-1855-0)
Supplement: Additional file 1: Table S1. — The sequences of DNA probes from An. sinensis Shanghai Chinese strain. (DOCX 15 kb) [file 13071_2016_1855_MOESM1_ESM.docx]

**Table S1**. The sequences of DNA probes from *An. sinensis* Shanghai Chinese Strain

> C15834 length=482

GTGTAGTAACCCCGTCCTAGGCGTCCTCCTCGAACCGATGCCAAACGGAGATCGGTGATCCCTTGTACTCGATCATCTTCCTCCACTGGTCCTCCATGGCCGGATTGGACTGGCGTGCCATCGGGCCCAGAAACACCGTACCGAGCCGTATCTTCTTGCCCACCTTACTCTTGGCCGCCAGACCTATCTTGATCGAAAGATTCTCCAGCGTTGTCTCGCCGCACAGTGGAACCACCAGCGTACAATCGTCCGCATTCCACCTGCTCGACAGGGCCGGTCGGAACGGTTCGGATTTCCAGCTGGCCGTTAGGATCTGGTGCTCGAGCACCGTCGTCTTCAGGTAGACCTTCTCCGCGATCTCCTCGAAACCCTCCTTCACCCGAATGCTGCAGCGCACCTTGGCGGTCGAGATCTTTAGGCACAGGCCGCGCTGGTTTTTGTCCTTGTTCTTGACGCTCCGCACGATCTTCCGGACGTCGAAG

>C20446 length=284

GACCTCGATGTCACGCAGTACCTTCACGCAGGCCGTGTCGCGCCCGGTCGCCACGAACTTGCAGTTCGCCCGGCAATCGTGGTTGATGTAGGCGGCCGGGCCGAGCCACAGCTGCGCGCAGTTCTTCCGGCAGCTGTACATCACGCTGAAGTCGTTCCGGCCCGGGTGCAGCAGCATCTCCTCCTCGCGCTCGCTCAGCTCGGCGATGCACCCGACCAGGCACTCGATCTTCTCGTTCTTCGCCCAGCGGCGCGTCGAGCAGATCTTCGCCCCCTTCTGGCCCT

>C07454 length=341

TGCCTCGCCGGTCGCGGGTATCCCACCGGCGATATCGCACCGGAAACCATTCCGACCGTGAAGCACGAGGTGCTGCGGTGCCTAACGGTGATCCACTCGAAGAACGCGCCGGAAGACGAGCTGCCGTATCCGTACCTGCGCAAGCTGCTCGAGTTTGATACGCGCGAAACGTTGAACGTCATTTCGCTCGCCTTCCAGGAGCGGGAGTTCAACGGCGAGCTGGGCCTGTCGCAGCGGCAGCGTATTATCAACATCCTGCTGAACGTGGTGACGCCGGATCGGGCCACCTGGTCGCAGGTCGGTGCCCTGCTCAACTTCATCGCGCAGCAGATTGCGTCCCG
